# Acceptability of placebo multiparticulate formulations in children and adults

**DOI:** 10.1038/s41598-018-27446-6

**Published:** 2018-06-15

**Authors:** Felipe L. Lopez, Punam Mistry, Hannah K. Batchelor, Joanne Bennett, Alastair Coupe, Terry B. Ernest, Mine Orlu, Catherine Tuleu

**Affiliations:** 1 School of Pharmacy, University College London, London, United Kingdom; 2 School of Pharmacy, Institute of Clinical Sciences, University of Birmingham, Birmingham, United Kingdom; 3 Pfizer Global R&D, Sandwich, Kent United Kingdom; 4 GlaxoSmithKline, Harlow, Essex United Kingdom

## Abstract

Patient acceptability is an important consideration in the design of medicines for children. The aim of this study was to investigate acceptability of multiparticulates in healthy children and adults. A randomised, single-blind acceptability testing was performed involving 71 children (4–12 years) and 61 adults (18–37 years). Each participant received three 500 mg samples of microcrystalline cellulose pellets administered on a medicine spoon with water at 5–10 minutes intervals. Acceptability was measured based on voluntary intake of the samples, facial expressions, ratings on hedonic scales and reported willingness to take multiparticulates everyday as a medicine. Multiparticulates were voluntarily swallowed by 92% of children and 100% of adults. However, palatability issues were identified, with emphasis on textural aspects. Grittiness perception received negative ratings on hedonic scales by 60% of children and 51% of adults. Researcher observations revealed that 72% of children and 42% of adults displayed negative facial expressions towards the samples. Children reported their willingness to take multiparticulates as a medicine in 30% of the cases, compared to 74% in adults. This study demonstrates that multiparticulates may be a suitable formulation platform for children and adults, although palatability concerns have been highlighted. Additional work is required to define acceptability criteria and to standardise methodologies.

## Introduction

Patient acceptability has been defined by the European Medicines Agency (EMA) as the ability and willingness of a patient and their caregiver to use and administer a medicine as intended^1^. Although not the only parameter that requires consideration, palatability is often regarded as one of the main elements influencing acceptability of oral medicines. Evaluation of patient acceptability should form an integral part of the pharmaceutical development studies and the Paediatric Investigational Plan (PIP), as recommended by the EMA in their Guideline on Pharmaceutical Development of Medicines for Paediatric Use^1^. There is also a moral obligation to evaluate acceptability of medicines to promote patient adherence and cost-effective treatments.

Consideration must be given to the selection of the most suitable formulation for the target population group^1^; however, fundamental knowledge about age-appropriateness of different dosage forms is still limited and fragmented^2^. Liquid medicines have been traditionally considered the most appropriate oral formulation for children, as they are easy to swallow by the patient and the dose can be titrated at the point of use. However, oral solid formulations are gaining support from regulatory authorities, based on their better stability profile, lower number of (potentially hazardous) excipients and suitability for taste-masking and controlled-release via film coating^1,3^. This paradigm shift in the selection of the most appropriate dosage form has been reinforced by recent studies supporting acceptability of solid medicines in children, including the youngest populations of preschool children and neonates^4–9^.

Multiparticulate formulations, in the form of pellets or beads, are composed of highly spherical granules of small diameter (typically below 1.5 mm) and narrow size distribution, usually prepared by fluidised bed technologies, including active layering and direct pelletisation^10^. Such formulations are suitable for controlled release and taste masking by means of film-coating technologies^11^, which could benefit patient compliance. Based on their multi-unit composition, multiparticulates offer higher potential for dose titration than conventional tablets and capsules^12^. For these reasons, multiparticulates are considered a flexible solid dosage form often proposed as an alternative to conventional solid and liquid formulations for children^3,13^. Despite the potential benefits of multiparticulates for paediatric drug delivery, evidence of patient acceptability is very scarce. Previous studies have investigated acceptability of multiparticulates in adult participants^14,15^, but acceptability studies in children are very limited^16^. More trials are required to investigate the impact of formulation factors (i.e. the effect of size of multiparticulates, presence or absence of film coating or amount of dosage units per dose) on patient acceptability.

Pharmaceutical regulatory bodies such as the EMA recommend to carry out palatability and acceptability testing of paediatric medicines in the targeted age group^1^. However, the pharmaceutical development of paediatric medicines traditionally relies on data from adult populations; although correlation between palatability and acceptability data in children and adults remains unknown. Despite the requirement to evaluate palatability and patient acceptability of paediatric medicines as part of the PIP, there is no regulatory guidance on how to perform such investigations^17^. Different study designs and outcome measures are used in current practice due to the lack of standardised methodology^18^. Visual Analogue Scales (VAS), Likert-type scales and hedonic scales have been often employed to evaluate specific sample attributes and/or overall acceptability^19,20^. In addition, the proportion of participants able to (safely and completely) swallow a pre-defined dose has been recently used as a simple measure of patient acceptability in various studies^4–6,9^.

The aim of this study was to evaluate acceptability of placebo multiparticulate formulations in healthy adults and children volunteers. Differences between both population groups in terms of formulation preferences and also regarding interpretation and use of data collection tools (i.e. questionnaires and scales) were investigated. Secondary objectives included to compare different methodologies for acceptability evaluation and to assess the effect of formulation factors on palatability and acceptability of multiparticulates. Researcher observations (such as the ability of participants to swallow the multiparticulates and facial expressions during sample intake) were complimented by and compared against subject-reported outcomes (including ratings on hedonic scales and willingness to take the sample everyday as a medicine). The formulation factors considered for the evaluation of sample preferences were the size of the multiparticulates (four sizes investigated) and the presence or absence of polymer coating.

## Results

### Demographics

A total of 132 participants were recruited, 71 children (4–12 years; median age = 7) and 61 adults (18–37 years; median age = 22). Since each participant received three multiparticulate samples, the total number of acceptability evaluations was 214 in children and 183 in adults. Children participants included 14 children between 4 and 5 years, 37 children between 6 and 8 years and 20 children between 9 and 12 years. Further details of the demographics have been provided in Supplementary Information. No significant association was found between the age of the children and any of the outcome measures evaluated, thus children were treated as a homogenous population for the purpose of data analysis.

### Acceptability of multiparticulates: comparison between children and adults

#### Success in swallowing the formulation

The proportion of participants who swallowed the complete dose of multiparticulates (as opposed to those who spat out the sample or refused it), was 92% in children and 100% in adults (Table 1). The sample was refused by five different children, two of whom refused two samples; the age of the children that refused the sample ranged from 5–8 years with a median of 7 years. The sample was spat out by eight children, one of whom spat out two samples; the children that spat out the sample ranged from 4–10 years with a median of 8 years. There were 10 occasions (5%) where children voiced resistance to taking the sample, and 20 occasions (10%) where children voiced disgust after sample administration. Other negative outcomes such as crying, screaming or vomiting were not observed in children before, during or after sample intake. No association was found between the age of the children and the frequency of negative behaviours towards the sample, including the sample being refused or spat out. No negative behaviours towards the samples were detected in adult participants.

**Table 1:** Acceptability of multiparticulates in children and adults based on researcher observations and subject-reported outcomes.

Evaluation criteria	Outcome	Children (n = 71, 213 evaluations)	Adults (n = 61, 183 evaluations)
Success in swallowing the formulation	Sample swallowed	197 (92.49%)	183 (100.00%)
	Sample spat out	9 (4.23%)	0 (0.00%)
	Sample refused	7 (3.29%)	0 (0.00%)
Sum of negative facial expressions^†^	0 - No discomfort	57 (27.67%)	107 (58.47%)
	1 - Light discomfort	74 (35.92%)	57 (31.15%)
	2 - Moderate discomfort	38 (18.45%)	13 (7.10%)
	3 - Considerable discomfort	16 (7.77%)	1 (0.55%)
	4 - Severe discomfort	21 (10.19%)	5 (2.73%)
Average rating on hedonic scales^†^	1 - Extremely liked	26 (12.62%)	20 (10.93%)
	2 - Liked	38 (18.45%)	52 (28.42%)
	3 - Neither liked/disliked	37 (17.96%)	78 (42.62%)
	4 - Disliked	55 (26.70%)	30 (16.39%)
	5 - Extremely disliked	50 (24.27%)	3 (1.64%)
Willingness to take the sample everyday^†^	Positive willingness	63 (30.58%)	135 (73.77%)
	Negative willingness	143 (69.42%)	48 (26.23%)

^†^Children refused the sample in seven occasions, thus their negative facial expressions and responses to hedonic ratings and willingness to take the sample every day were not recorded, and results were calculated based on a total of 206 evaluations instead of 213.

#### Researcher observations of facial expressions

Negative facial expressions were recorded as an indicator of participant’s discomfort and dislike. The most commonly observed facial expression was ‘pursed lips’ (with 57% of children and 34% of adults displaying this behaviour), followed by ‘nose wrinkle’ (30% and 10%), ‘brow bulge’ (26% and 9%) and ‘eyes squeezed’ (21% and 5%, respectively). Comparing the two population groups, the sum of negative facial expressions was consistently higher in children than adults (p = 0.003), which suggests better acceptance of the samples by adults. Children showed at least one negative facial expression in 72% of the occasions, whereas this measure accounted for 42% in adults.

#### Participants ratings on hedonic scales

**Figure 1:**
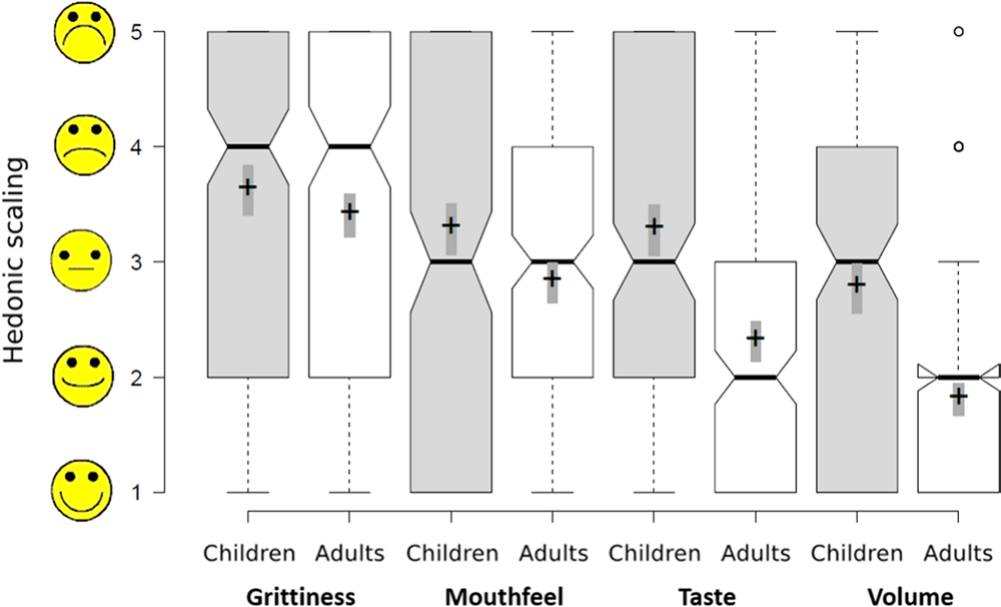
Ratings of sample attributes in hedonic scales (where 1 represents ‘extremely liked’ and 5 represents ‘extremely disliked’) by population group. This graph shows pooled data from all different samples (4 particle sizes, coated/uncoated). Centre lines show the medians, box limits indicate the 25th and 75th percentiles, notches represent the 95% confidence interval of the median and outliers are denoted by dots; crosses represent sample means and bars indicate 95% confidence intervals of the means.

Four different criteria were evaluated using 5-point hedonic scales: grittiness, mouthfeel, taste and sample volume. Overall, grittiness was the most negatively rated attribute, with a median value of 4 in both adults and children (Fig. 1). Grittiness perception received negative hedonic scores (i.e. hedonic rating = 4–5) by 60% of children and 51% of adults. On the contrary, the most favourably rated item was sample volume, with a median of 2 and 3 in adults and children, respectively. Overall, ratings of palatability descriptors were significantly worse in children than in adults (p < 0.001), particularly for taste and sample volume. This indicates relatively lower acceptance of multiparticulates in children, in agreement with the previous findings based on researcher observations of facial expressions. Adult participants provided average hedonic ratings to the four palatability attributes in the neutral-positive range of the scale in 82% occasions, whereas this value was 49% in children.

Participants’ spontaneous descriptions of the samples support the findings of hedonic scales, samples were often described as ‘tasteless’ or as having ‘no flavour’; however, the feeling in the mouth was found to be ‘gritty’ and ‘sandy’. The feedback provided by children often denoted the lack of flavour as a negative aspect of the formulation, e.g. “it was horrible because it had no flavour” or “if it had a flavour it would be nice”, which could explain why taste received negative ratings in hedonic scales. Moreover, children often employed the term “gritty” as a negative attribute of taste (instead of mouthfeel), e.g. “I hated the gritty taste” or “the taste was bad because it was gritty”, which could further explain the negative ratings given to taste in contrasts with ratings provided by adults. A significant association was found between each possible pair of palatability descriptors in both children and adults (Chi-squared test for association, p < 0.001 for each pair combination). This suggests the existence of a ‘halo effect’, by which participants’ responses to one attribute were influenced by their opinion and responses to another attribute^21^. For example, when the mouthfeel of the sample was disliked, the taste of the sample would tend to be disliked as well. Participants voluntary feedback also highlighted the novelty of the formulation, e.g. “I have never tasted something like this before” or “because it was the first time to try it, mouthfeel was strange” (comments made by adult participants).

#### Willingness to take the sample everyday if it was a medicine

The proportion of participants willing to take the sample every day if it was a medicine was calculated as a predictive measure of future and repeated acceptability, which is likely to influence adherence. Overall, children reported their willingness to take the sample every day in 63 occasions (30%), their response being negative in most cases (143, 70%). On the contrary, a larger proportion of adults stated that they would take the sample every day (135, 74%) compared to those who would not be willing to take it (48, 26%). A significant association was found between ratings to palatability descriptors and the willingness of participants to take the sample every day. This association was stronger for grittiness, mouthfeel and taste (p < 0.001 in all cases) than it was for sample volume (p = 0.004). This means that those patients who rated palatability positively would tend to be more willing to take the sample every day, as it could be expected. Interestingly, the willingness to take the sample in adults (74%) was aligned with the measure of acceptability based on hedonic ratings (82%); however, children reported to be willing to take the sample every day (30%) less frequently than expected based on hedonic ratings (49%).

### Effect of formulation factors on palatability and acceptability

Looking at the proportion of participants who swallowed the formulations (as opposed to those who refused or spat out the sample), children accepted multiparticulates in 92% of the occasions and adults accepted multiparticulates in all cases. Therefore, analysis of the effect of size and coating on acceptability was impractical. Based on this outcome measure, multiparticulates were well accepted regardless of the formulation properties. Similarly, based on researcher observations of negative facial expressions, no significant differences between different multiparticulate sizes and between coated or uncoated samples were found in either children (p = 0.923 and p = 0.800 for size and coating effects, respectively) or adults (p = 0.551 and p = 0.795, respectively).

**Figure 2:**
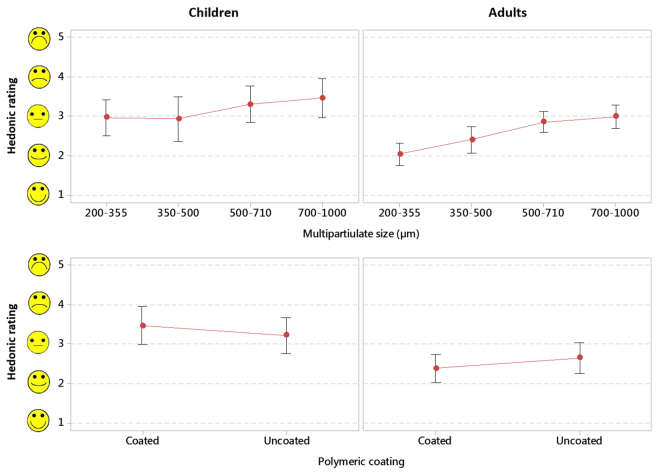
Ratings of sample attributes in hedonic scales as a function of multiparticulate size and presence of polymeric coating, by population group. Markers represent the combined average rating of grittiness, mouthfeel, taste and sample volume and bars represent the 95% confidence interval.

**Figure 3:**
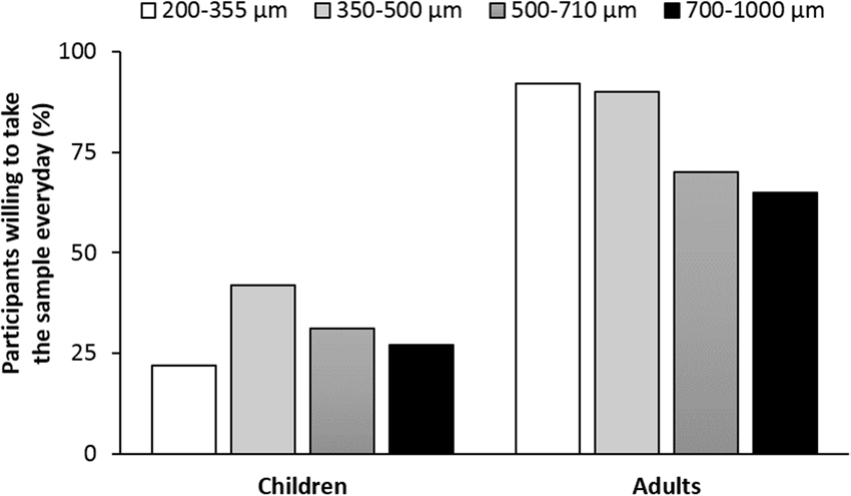
Proportion of participants reporting willingness to take multiparticulates everyday if it was a medicine.

Evaluation of their ratings of sample attributes on hedonic scales, adults perceived larger particles as being ‘grittier’ (p < 0.001) and thus showed preference for smaller particles (Fig. 2). Although there was no significant evidence of size effect in children (p = 0.078), the larger two sizes received more negative scores on average than the lower two sizes. There was no evidence of a coating effect on hedonic scores in either adults (p = 0.276) or children (p = 0.466). Particle size had a significant effect on the reported willingness to take the sample everyday by adult participants, who showed preference for smaller particles (p = 0.039), in agreement with their responses using hedonic scales (Fig. 3). In the case of children, particle size had no clear effect on the willingness to take the sample everyday (p = 0.112). The effect of coating on the reported willingness to take the sample everyday was trivial in both children and adults (p = 0.407 and p = 0.116, respectively).

Since each participant received three sequential samples, the effect of administration order on outcome measures was investigated and no significant effect was found.

### Water consumed and residual multiparticulates in the mouth

**Figure 4:**
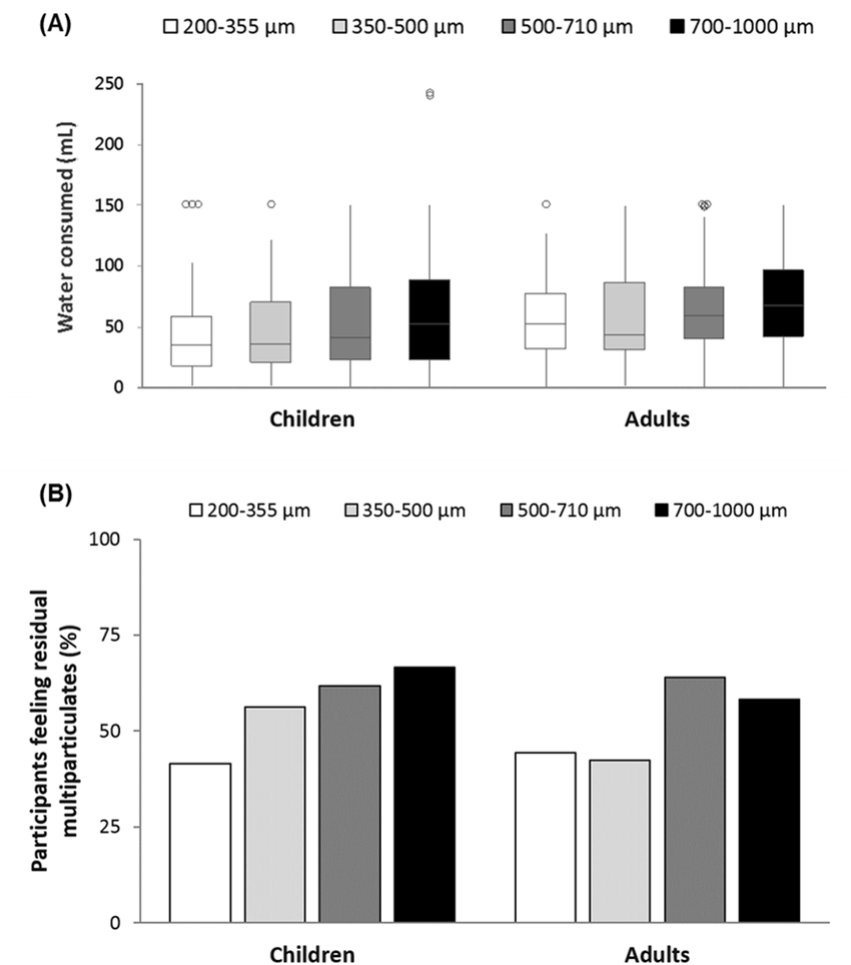
(**A**) Volume of water consumed as a function of multiparticulate size, where centre lines show the medians, box limits indicate the 25th and 75th percentiles and outliers are denoted by dots. (**B**) Proportion of participants that reported they could still feel residual multiparticulates in their mouth after sample intake, as a function of particle size.

In addition to the small volume of water used to pre-disperse multiparticulates on the dosing spoon (approximately 3 mL), participants had free access to spring water to complete sample intake. Children consumed 51 mL of water on average (median = 40 mL, min = 0 mL, max = 242 mL), whereas adults consumed 63 mL on average (median = 56 mL, min = 0 mL, max = 150 mL). The volume of water consumed was comparable between formulations; although there was a trend of increasing water consumed with increasing multiparticulate size (Fig. 4).

After rinsing their mouth with water, participants were asked if they could still feel the multiparticulates in their mouth. Overall, adults reported that they could still feel particles in their mouth in 97 occasions (53%), whereas children reported they could feel remaining particles in 116 occasions (56%). In general, the reported feeling of residual multiparticulates in the mouth increased with increasing particle size (Fig. 4), although this trend was not statistically significant in either children or adults (p = 0.057 and p = 0.106, respectively).

## Discussion

Results of this trial indicate acceptability of multiparticulates in terms of ability to use as required and swallow the formulation. Adults took the multiparticulate samples in all occasions, whereas children took the sample in 92% of the occasions. Although the sample was refused or taken but then spat out by children in some instances (3% and 5%, respectively), no undesirable effects such as vomiting or choking with the sample were observed or reported. However, researcher observations and participant-reported outcomes denoted some level of discomfort and sample dislike by the participants of the study. Negative facial expressions towards the samples were displayed by 72% of children and 42% of adults. These results were in line with the participant-reported outcomes, since 60% of children and 51% of adults provided negative hedonic scores to grittiness perception. The willingness to take the sample everyday if it was a medicine was determined to be 30% in children and 74% in adults, based on participant-reported outcomes. Overall, adult participants showed broader acceptance of the samples than children, as consistently shown by each of the different outcome measures.

Participants discomfort and sample dislike could be ascribed to the ‘gritty’ feeling in the mouth produced by multiparticulates, as demonstrated by negative hedonic ratings of grittiness perception and spontaneous verbal judgment of the samples. These findings are supported by previous research in adults^14,15^. To some extent, the negative ratings of grittiness could be influenced by the lack of exposure to this type of formulation. Neophobia, a predisposition to avoid new products, is well established in humans^22^. Future studies looking at repeated exposure could capture if there are changes in participants’ perceptions of multiparticulates over time, especially in terms of grittiness. In addition, 56% of children and 53% of adults reported a feeling of residual multiparticulates in the mouth after sample intake. A long-lasting sensation of rough mouthfeel has also been reported in a previous study in adults^15^. This was an important finding given that multiparticulates are often intended for taste-masking and a prolonged residence time in the mouth might put the integrity of the coating at risk (by incentivising dissolution and/or chewing).

In terms of sample preferences, children showed no significant preference for any sample, although the larger two sizes of multiparticulates (>500 µm) obtained more negative hedonic ratings than the smaller two sizes; adults showed clear preference for smaller multiparticulates, based on participant-reported outcomes. These findings are consistent with previous studies which showed increased grittiness perception with increased size of multiparticulates^14,15^. The presence of film coating on the multiparticulates did not seem to influence acceptability of the samples in either children or adults. A smooth polymeric film coating could reduce surface roughness, which could have a positive impact on palatability by reducing grittiness perception and facilitating swallowing. However, a previous study of placebo mini-tablets (2 mm in size) found no significant differences in acceptability between coated and uncoated minitablets^6^, in line with the present study. It should be noted that coating can be expected to have a critical impact on patient acceptability of drug-loaded formulations by providing taste-making.

The samples used in this study contained a large amount of multiparticulates (500 mg in 3 ml of water) and were neutral tasting (i.e. no sweeteners or flavours were added). Development of a suitable vehicle for the administration of multiparticulates could have a beneficial impact on the final formulation via masking the presence of particles and/or adding a flavour to make it more palatable. However, co-administration of medicines with food or drinks also poses safety concerns, such as poor control over dose intake and impact on drug’s bioavailability^23^. Typical vehicles recommended for sprinkle products include apple sauce and yogurt that provide both flavour and viscosity to improve palatability and ease of swallowing. Investigation into swallowing aids for the administration of oral solid formulations have been the focus of previous research, with some products already in the market in the form of sprays, pastes or jellies^24–26^.

The lack of standardised methodology for acceptability testing is a barrier for the development of paediatric medicines. Most studies reported in the scientific literature have employed questionnaires accompanied by facial/visual analogue scales to measure acceptability^18^, although there is no clear evidence of the validity and reliability of participant-reported outcomes in children below 12 years^27,28^. In the present study, the ability of participants to independently rate each item on the hedonic scales can be questionable, since ratings of different items were found to be associated with each other (in both population groups). Moreover, the interpretation of the meaning of each item could be inconsistent between participants; for instance, children often referred to the term ‘grittiness’ as an attribute of taste instead of texture in their spontaneous judgement of the samples. These phenomena can be expected, especially from untrained assessors, and a number of studies have addressed these issues in the past^29–31^.

Despite the limitations of participant-reported outcomes, these provided better discrimination between samples than researcher observations. In addition, participant-reported outcomes offered individual evaluation of a range of sample attributes, including taste, texture and sample volume (i.e. analytical evaluation); whereas researcher observations can only be used to obtain a global judgement of the samples (i.e. synthetic evaluation). Nevertheless, researcher observations would be preferred when the use of participant-reported outcomes is impractical (e.g. patients who have cognitive impairment or are unable to communicate). As shown in this trial, complimentary outcome measures may be used in combination; the ability to swallow the formulation focused on a single administration whereas the others could provide insights into the long-term acceptability of the formulation.

This study was conducted in healthy subjects, in a controlled environment, under supervision of the research team; this allows no definitive conclusion of the safety and acceptability of multiparticulates in ill children or administered by lay people in their home environment but may provide an indication of the suitability and acceptability of multiparticulates. Results for the willingness of participants to take multiparticulates every day as a medicine require cautious interpretation; although this outcome measure could provide an indication of future acceptance, results were based on opinions of healthy participants after a single administration. Nevertheless, previous research suggests that participant-reported attitudes towards taking a medication or to continue with a therapeutic regime are good predictors of patient adherence^32,33^. The study should be extended to younger children based on the positive results in terms of ability to swallow the formulation and safe administration.

## Conclusion

This trial suggests that multiparticulates could be used as a suitable formulation platform for the administration of medicines to adults and children (four-year-old and above), although palatability might be a barrier to patient acceptability due to gritty mouthfeel. As demonstrated in this trial, a simple measure of participants ability to swallow the sample might not be sufficient to evaluate patient acceptability, understood not only as ability but also willingness to take the product. Despite of the vast number of participants that accepted to take the sample, researcher observations and participant-reported outcomes showed a significant proportion of participants’ discomfort and dislike. Researcher observations of facial expressions and behaviours, participants’ hedonic ratings and spontaneous verbal judgement of the samples provided a valuable insight into patient acceptability and sample preferences. More trials are required to develop standardised methodology for palatability and acceptability testing.

## Methods

### Study design

A randomised, single-blind, patient acceptability study was conducted in children (inclusion criteria: healthy volunteer, 4–12 years old) and adults (inclusion criteria: healthy volunteer, 18–40 years old). The study was performed in accordance with the ethical principles that have their origin in the Declaration of Helsinki. The study in children was approved by the University of Birmingham Research Ethics Committee (ERN_15–1028) and took place in a designated room at Thinktank Science Museum (Birmingham, United Kingdom). Participant information leaflets were distributed to potential participants on admission to the science museum. Parents/carers received a detailed information sheet and children received an information leaflet designed to be appropriate for children. They were given adequate time to read and consider the information provided and to ask questions before a parent and/or legal guardian signed informed consent for study participation. The study in adults was approved by the University College London Research Ethics Committee (6062–001) and was conducted in a designated room at the UCL School of Pharmacy. Participant information leaflets were distributed via email to potential participants, including University staff and students. All adult participants received a detailed information sheet and provided written consent to participate in the study.

**Figure 5:**
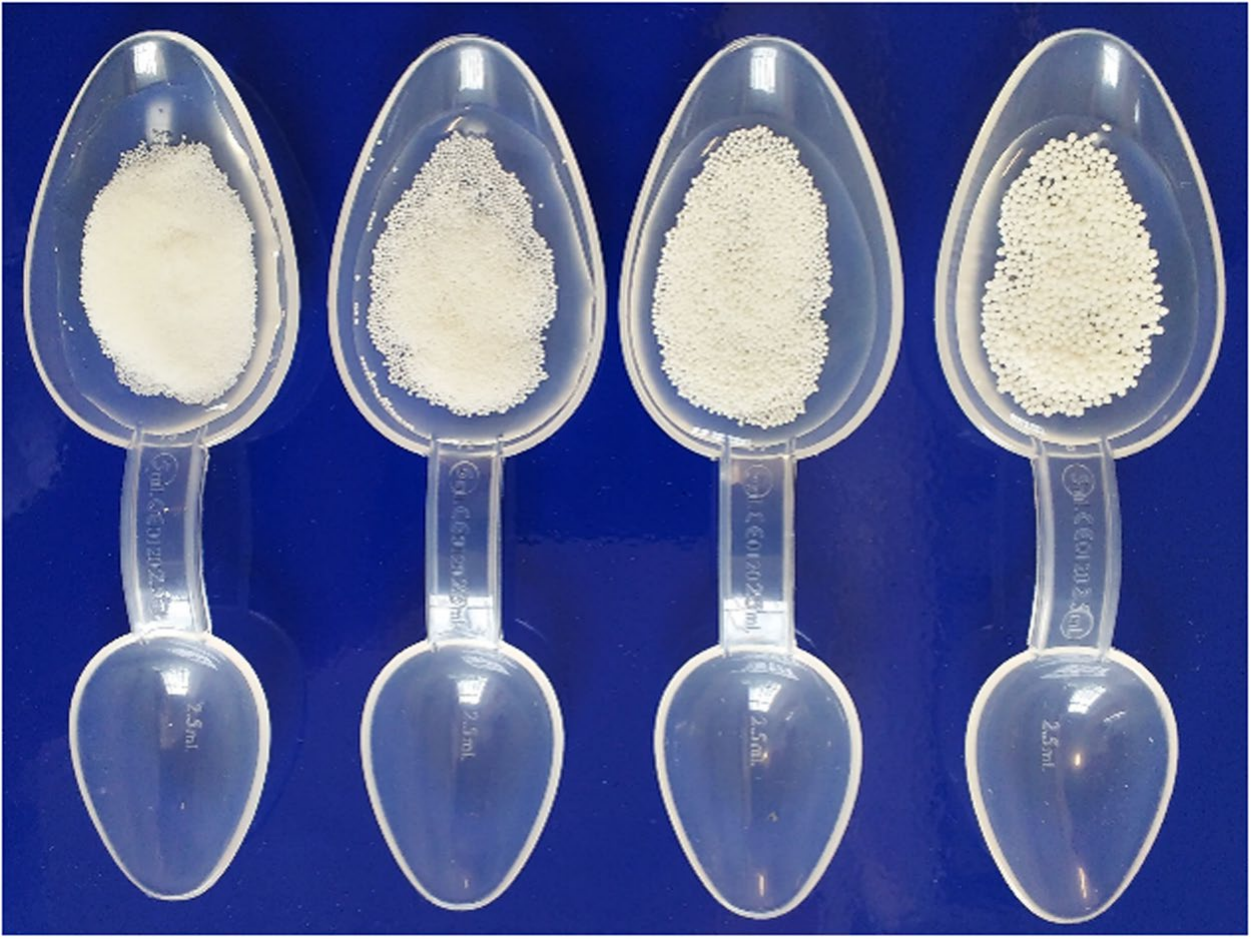
Samples of 500 mg of placebo multiparticulates dispersed in approximately 3 ml of spring water on a medicine dosing spoon. The particle size of the multiparticulates varies, from left to right: 200–355, 350–500, 500–710 and 700–1000 μm.

Microcrystalline cellulose pellets (Cellets®, Pharmatrans Sanaq, Basel, Switzerland) were used as model placebo multiparticulates. Coated versions of the multiparticulates were produced under good manufacturing practices by Pfizer (Sandwich, United Kingdom) by coating Cellets® with a Kollicoat® Smartseal 30D polymeric film, typically used for taste-masking. Both coated and uncoated multiparticulates were non-disintegrating in the mouth. The sizes investigated included those at 200–355, 350–500, 500–710 and 700–1000 µm. Each participant received three 500 mg samples of placebo multiparticulates administered on a medicine spoon with approximately 3 ml spring water, added immediately before administration (Fig. 5). Participants had free access to additional spring water as required to complete sample intake. Samples were self-administered by the participants of the study, except for a few cases where the sample was administered to the child (with the child’s verbal consent) by one of the investigators to avoid spillages. An interval of 5–10 minutes was maintained between samples to minimise subject discomfort and carry over effect.

The study was divided in two phases, with four sessions per phase to ensure a balanced allocation ratio (Table 2). Phase 1 was dedicated to the evaluation of the effect of particle size and thus each participant received 3 samples of varying particle size in a randomised order (all samples were polymer coated for this part of the study). Phase 2 was dedicated to the evaluation of the effect of coating and thus each participant received two samples of identical particle size, one coated and one uncoated in a randomised order, plus an additional uncoated sample. Based on power calculations assuming parametric unimodal distribution, significance (α) of 0.05 and 90% power; a sample size of 14 would show the difference between two samples where the difference was one point and the standard deviation was also one point on a 5-point scale.

**Table 2:** Dosing schedule for the evaluation of multiparticulates.

Sample		Phase 1 (size effect)				Phase 2 (coating effect)			
Size (µm)	Coating	S1	S2	S3	S4	S5	S6	S7	S8
200–355	Coated	1	3		2	1			
350–500	Coated	2		3	1		2		
500–710	Coated		2	1	3			2	
700–1000	Coated	3	1	2					1
200–355	Uncoated					2	3		
350–500	Uncoated					3	1		
500–710	Uncoated							1	3
700–1000	Uncoated							3	2

Numbers indicate the order in which samples were administered in each of the eight sessions (S1-S8).

### Data collection

Patient acceptability can be influenced by several factors such as palatability, swallowability and ease of administration. Consequently, a range of outcome measures were employed to gather information about the different factors related to acceptability.

#### Researcher observations

Each participant was observed by two researchers, who evaluated facial expressions and behaviours prior to, during and post sample intake using a 12-point tick chart (Table 3). Spontaneous verbal judgement of the samples was also recorded in researcher observation sheets. The proportion of participants who swallowed the complete dose of multiparticulates (as opposed to those who spat out the sample or refused it) was determined, as used in previous studies with mini-tablets^4–6,9^. In addition, the sum of negative facial expressions (0–4) was calculated to investigate participants’ affective response and sample preferences. A value of 0 was regarded as indicating no discomfort, 1-light discomfort, 2-moderate discomfort, 3- considerable discomfort, 4-severe discomfort. Negative facial expressions recorded during sample intake are expected to be a good indicator of sample dislike, based on previous research with school-aged children^34^.

**Table 3:** Researchers’ observations 12-point tick chart for assessing negative facial expressions and behaviours of participants prior to, during and after sample intake.

Behaviours during/prior to administration	Behaviours immediately after administration	Negative facial expressions
Refuses test sample	Spits out test sample	Pursed lips
Voices resistance	Voices disgust	Nose wrinkle
Cries/screams	Cries	Brow bulge/lower (frown)
Requires physical restraint	Vomits	Eyes squeezed shut

#### Participant-reported outcomes

Participant-reported outcomes were collected using a paper-based structured questionnaire that was filled in by the participants of the study immediately after sample intake. Participants could also provide a voluntary written description of the sample, which was used to facilitate interpretation of results. Samples were evaluated using 5-point hedonic scales for four different attributes: grittiness (explained to participants as “you can feel ‘bits’ in the sample”), sample volume (“the amount that you had to take”), overall mouthfeel (“how the sample felt in your mouth”) and overall taste. Results of hedonic scales were assigned numerical values from 1 (extremely liked) to 5 (extremely disliked) for the purposes of data analysis and the average hedonic rating to the four sample attributes was calculated. Samples with average hedonic rating of 1–3 were deemed not aversive, thus palatable. After completion of hedonic ratings, participants answered the following question: “If this was a medicine, would you be willing to take it every day?”. The proportion of participants that responded positively to this question was calculated. The total volume of water consumed for each sample was calculated by providing free access to water in cups with a pre-measured volume (150 ml) and then measuring the volume of water left in the cups. The proportion of participants that reported that they could still feel the bits in their mouth after sample administration was determined.

### Statistical analysis

Data from researcher observations and participant-reported outcomes was treated as categorical data; differences between population groups and association between outcome measures were assessed by Pearson’s Chi-squared test with 95% confidence interval. The effect of formulation factors on acceptability ratings (i.e. sum of facial expressions and ratings on hedonic scales) was assessed by comparing mean scores using ANOVA techniques. Size effect was estimated by analysis of subjects in Phase 1 only, as this enables a better within subject comparison of sizes. Coating effect estimate was limited to analysis of subjects in Phase 2 (after exclusion of test sample 3), such that the coating effect is purely within subject. The volume of water consumed was treated as a non-normally distributed continuous variable (based on Kolmogorov–Smirnov test) and differences between samples and between population groups were assessed by Kruskal-Wallis test.

### Data availability statement

The datasets generated and analysed during the current study are available from the corresponding author on reasonable request.

## Electronic supplementary material

Supplementary Information
