# Supplementary material for: Acceptability of placebo multiparticulate formulations in children and adults
Source: Sci Rep. 2018 Jun 15;8:9210. doi: 10.1038/s41598-018-27446-6 (PMC6003938; doi:10.1038/s41598-018-27446-6)
Supplement: Supplementary file 1 — Supplementary Information [file 41598_2018_27446_MOESM1_ESM.docx]

**Acceptability of placebo multiparticulate formulations in children and adults**

Felipe L. Lopez^a^, Punam Mistry^b^, Hannah K. Batchelor^b^, Joanne Bennett^c^, Alastair Coupe^c^, Terry B. Ernest^d^, Mine Orlu^a^, Catherine Tuleu^a,^*.

Affiliations: ^a^School of Pharmacy, University College London, London, United Kingdom. ^b^School of Pharmacy, Institute of Clinical Sciences, University of Birmingham, Birmingham, United Kingdom. ^c^Pfizer Global R&D, Sandwich, Kent, United Kingdom. ^d^GlaxoSmithKline, Harlow, Essex, United Kingdom.

* Corresponding author: Catherine Tuleu, UCL School of Pharmacy, 29-39 Brunswick Square, London, WC1N 1AX, United Kingdom, [c.tuleu@ucl.ac.uk], +44(0)20 7753 5857.

**Supplementary information**

Demographic characteristics of the study participants.

| **Session** | **Children** | | | **Adults** | | |
| --- | --- | --- | --- | --- | --- | --- |
|  | **N** | **Age (years)**  **Ave ± SD** | **Age (years)**  **Min. - Max.** | **N** | **Age (years)**  **Ave ± SD** | **Age (years)**  **Min. - Max.** |
| 1 | 9 | 6.6 ± 1.7 | 4 - 9 | 6 | 25.3 ± 5.2 | 21 - 35 |
| 2 | 13 | 8.2 ± 2.2 | 5 - 12 | 12 | 23.0 ± 5.3 | 19 - 37 |
| 3 | 8 | 6.6 ± 2.2 | 4 - 10 | 8 | 20.6 ± 3.0 | 18 - 27 |
| 4 | 7 | 7.0 ± 2.1 | 5 - 11 | 7 | 20.7 ± 2.1 | 18 - 25 |
| Phase 1 total | 37 | 7.2 ± 2.1 | 4 - 12 | 33 | 22.4 ± 4.5 | 18 - 37 |
| 5 | 8 | 7.1 ± 2.3 | 4 - 10 | 7 | 23.6 ± 4.9 | 19 - 30 |
| 6 | 7 | 8.7 ± 2.0 | 6 - 11 | 7 | 24.7 ± 4.3 | 20 - 32 |
| 7 | 12 | 6.8 ± 1.5 | 4 - 9 | 6 | 26.5 ± 4.3 | 24 - 35 |
| 8 | 7 | 6.9 ± 2.4 | 4 - 11 | 8 | 26.6 ± 4.9 | 20 - 34 |
| Phase 2 total | 34 | 7.3 ± 2.1 | 4 - 11 | 28 | 25.4 ± 4.6 | 19 - 35 |
| Grand total | 71 | 7.2 ± 4.0 | 4 - 12 | 61 | 23.7 ± 4.7 | 18 - 37 |

The ages of the volunteers that participated in the study are shown in the histograms below:

Figure 1. Histogram of age for adult participants.


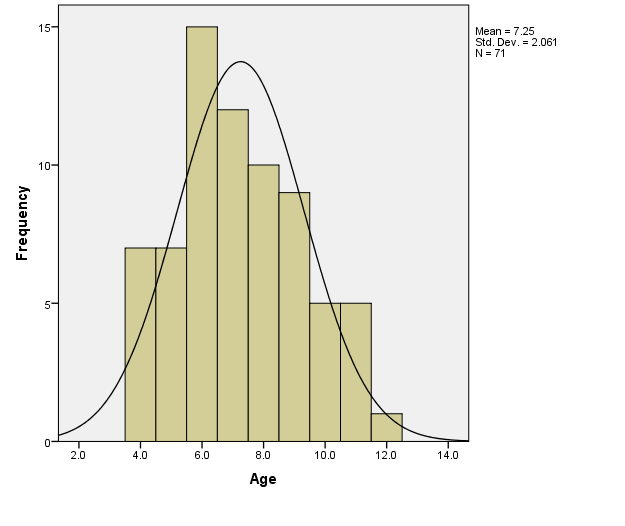


Figure 2. Histogram of age for children participants.
